# Targeting BCL-2 regulated apoptosis in cancer

**DOI:** 10.1098/rsob.180002

**Published:** 2018-05-16

**Authors:** Kirsteen J. Campbell, Stephen W. G. Tait

**Affiliations:** 1 Cancer Research UK Beatson Institute, University of Glasgow, Garscube Estate, Switchback Road, Glasgow, G61 1BD, UK; 2 Institute of Cancer Sciences, University of Glasgow, Garscube Estate, Switchback Road, Glasgow, G61 1BD, UK

**Keywords:** BCL-2 family, apoptosis, BAX/BAK

## Abstract

The ability of a cell to undergo mitochondrial apoptosis is governed by pro- and anti-apoptotic members of the BCL-2 protein family. The equilibrium of pro- versus anti-apoptotic BCL-2 proteins ensures appropriate regulation of programmed cell death during development and maintains organismal health. When unbalanced, the BCL-2 family can act as a barrier to apoptosis and facilitate tumour development and resistance to cancer therapy. Here we discuss the BCL-2 family, their deregulation in cancer and recent pharmaceutical developments to target specific members of this family as cancer therapy.

## 1. Introduction

Apoptosis is a form of regulated cell death that is triggered in response to developmental cues or cellular stress. This selective cell suicide plays an essential role in numerous physiological and pathological processes including development, immunity and disease where the elimination of damaged or superfluous cells helps to ensure organismal health [1].

There are two apoptotic pathways—the extrinsic pathway (activated by ligand engagement of cell surface death receptors) and the intrinsic (mitochondrial) pathway. This review focuses on the BCL-2 family of proteins that regulate activation of the intrinsic apoptotic pathway in response to cellular stresses such as DNA damage, γ-irradiation, oncogene activation and growth factor withdrawal.

Recent pharmaceutical advances have allowed the specific targeting of protein–protein interactions in the BCL-2 (B-cell lymphoma 2) family [2–4]. Early clinical results in haematological cancers show considerable promise. This review will summarize apoptotic pathway regulation by the BCL-2 family, their perturbation in cancer and utility as therapeutic targets.

## 2. The BCL-2 family

**Figure 1.**
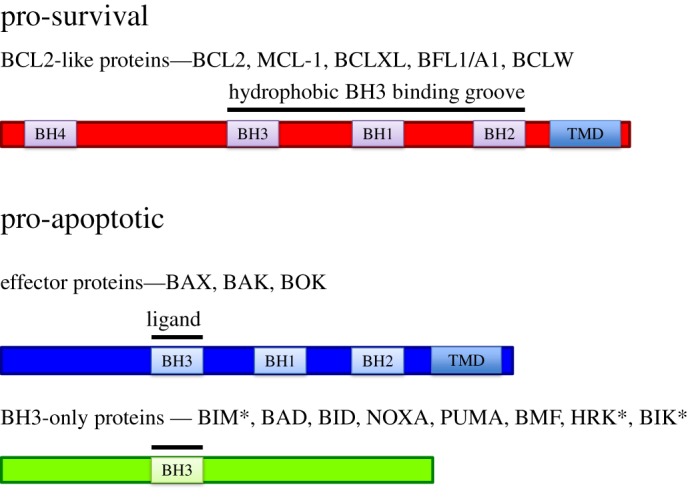
The BCL-2 family is composed of pro-survival and pro-apoptotic proteins. BCL-2 family members show sequence homology to BCL-2 in one or more BH (BCL-2 homology) domain. These proteins can be divided into pro-survival and pro-apoptotic proteins. Within the pro-apoptotic members there is a further subdivision between the multi-BH domain containing effector proteins and those proteins whose only region of homology to BCL-2 is BH3 (known as BH3-only proteins). Membrane insertion is mediated by transmembrane domains (TMD) present in pro-survival, effector and some BH3-only proteins (*BIM, BIK and HRK).

The founder member, BCL-2, was first identified through chromosomal mapping in follicular lymphoma where constitutive BCL-2 expression is driven from the immunoglobulin locus by the t[12;18] translocation [5–7]. Unlike the cell growth and proliferative functions of other known oncoproteins at that time, BCL-2 was found to facilitate oncogenesis through cell death resistance [8,9]. In the following years over 15 proteins have been added to this family, each containing one or more BCL-2 homology (BH) domain and functional studies have allowed grouping into three classes (figure 1).

A clear division in the family exists between members that function to prevent apoptosis (pro-survival or anti-apoptotic) and those that induce apoptosis (pro-apoptotic). The pro-apoptotic BCL-2 family members can be further divided into the multi-BH-domain effector proteins (containing BH1, BH2 and BH3 domains) and BH3-only proteins (only region of homology to BCL-2 is BH3) (figure 1).

Physical interaction between pro-survival and pro-apoptotic family members can buffer the cell against the onset of mitochondrial-mediated apoptosis. Structural studies have revealed that BH1, BH2 and BH3 regions together form a hydrophobic pocket that can be filled by the amphipathic α-helical BH3 domain of pro-apoptotic BCL-2 proteins [10,11]. The balance of this interaction ensures appropriate apoptotic regulation in response to development cues and cellular stresses. In a simple model, when pro-survival proteins predominate, apoptosis is held in check: when pro-apoptotic proteins predominate, apoptosis is triggered. However, localization and conformation of BCL-2 proteins is also important in regulation of activity.

## 3. Pro-survival BCL-2 proteins

**Figure 2.**
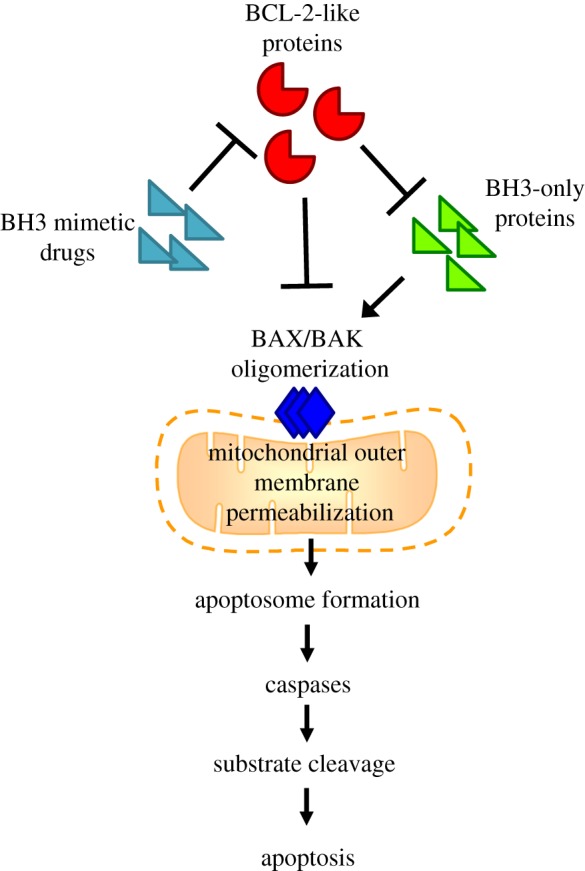
BCL-2 family interactions regulate mitochondrial outer membrane permeabilization (MOMP). Interaction between pro-survival and pro-apoptotic BCL-2 proteins sets a threshold for activation of apoptosis. BCL-2-like pro-survival proteins inhibit BAX/BAK activation whereas BH3-only proteins promote BAX/BAK oligomerization. Drugs mimicking the action of BH3-only proteins indirectly lead to BAX/BAK activation. This allows MOMP, apoptosome formation and subsequent caspase activation and apoptosis.

Pro-survival proteins such as BCL-XL, MCL-1, BFL1 (A1 in mouse) and BCL-W contain multiple regions of homology to BCL-2 (figure 2). Each of these proteins is found in many cell types/tissues and co-expression of multiple pro-survival proteins often occurs. The relative expression levels can vary in a cell type and developmental manner and are perhaps best characterized in the haematopoietic system. For example, dynamic patterns of pro-survival BCL-2 gene expression occurs during B lymphocyte development with *Bclx* being expressed early in B-cell development, *Mcl1* and *Bcl2* expression generally increasing with B-cell maturity and *A1* levels peaking in the intermediate stages [12]. In this way, different pro-survivals play key roles at distinct stages of development.

Levels of pro-survival BCL-2 proteins can also be regulated by protein turnover. BCL-2 and BCL-XL are relatively stable proteins (e.g. the half-life of BCL-2 approx. 20 h) [13]. By contrast, MCL-1 and A1 protein turnover is constitutive through polyubiquitination and proteosomal degradation (reflected in their short half-lives approx. 30 and approx. 15 min, respectively) [14–17]. In this way, levels of MCL-1 and A1 help facilitate dynamic responses to cell death stimuli.

Gene deletion studies have revealed essential and non-redundant roles of pro-survival BCL-2 proteins in mice. While embryogenesis proceeds normally in the absence of *Bcl2*, deficient mice show postnatal growth retardation, premature greying, apoptotic involution of spleen/thymus and succumb to early mortality through polycystic kidney disease with altered renal cell differentiation and elevated apoptosis [18]. Young *Bcl2^−/−^* mice have normal haematopoietic populations, but this is not sustained, with notable loss in peripheral B and T lymphocyte populations [18,19]. These phenotypes can be reversed by loss of one or two *Bim* alleles (encoding a BH3-only pro-apoptotic member of the BCL-2 family), indicating that sequestration of BIM is the major function of BCL-2 [20].

Deletion of *Bclx* is lethal around embryonic day 13 with extensive neuronal and haematopoietic apoptosis [21], loss of *Bim* can rescue the haematopoetic but not neuronal phenotype in *bclx* null embryos [22]. In adult mice, acute deletion of *Bclx* is tolerated (animals were followed for 1 month) but still resulted in severe anaemia, consistent with *Bclx* being required for reticulocyte survival [23]. In contrast to embryogenesis, loss of *bim* could not restore erythropoiesis in adults [23].

Deletion studies of *A1* had been incomplete until recently when knockout of all three functional isoforms of A1 in mice was achieved. Surprisingly, A1 function seems largely redundant, with only minor impact on subsets of cells in the haematopoietic system [24]. The function of BCL-W also appears mostly dispensable for normal development and health, but *Bclw-*deficient males are infertile due to a defect in spermatogenesis [25,26].

Across the BCL-2 family the phenotype of the *Mcl1* knockout mouse is most severe. *Mcl1* deficiency results in early lethality at pre-implantation stage, but these blastocysts showed no evidence of increased apoptosis [27], providing a suggestion of a non-apoptotic role for MCL-1. Conditional deletion studies in the adult mouse have also revealed an essential role for MCL-1 in numerous cell types, including T and B lymphocytes [28], haematopoietic stem cells [29], cardiomyocytes [30,31], hepatocytes [32], neuronal progenitors [33] and neutrophils, but not macrophages [34,35], mammary epithelium or megakaryocytes [36,37].

Interestingly, while MCL-1 deficiency alone in megakaryocytes had no impact, when combined with loss of *Bclx* this caused embryonic or pre-weaning lethality, which is also far more dramatic than the impaired platelet shedding phenotype that is found with loss of just *Bclx* [36,37]. This could be rescued by co-deletion of *Bax/Bak* [37], but clearly illustrates the co-dependence of certain cell types on multiple pro-survival proteins. This co-dependence has been shown in elegant detail in a recent study of immune populations where multiple pro-survival proteins were targeted by genetic and pharmacologic methods [38]. Therefore, while gene knockout studies have given much insight into cell types in which individual pro-survival proteins have a dominant role, there has probably been an underestimation of the extent of their contribution to cell survival in many other cell types. Indeed, the sum effect of all pro-survival proteins present may be more important for survival than expression levels of an individual protein. This is an important consideration for therapeutic targeting of pro-survival BCL-2 proteins and minimization of damage to normal tissues.

## 4. Pro-apoptotic BCL-2 members

Pro-apoptotic BCL-2 proteins fall into two sub-classes (figure 2). BH3-only proteins such as BIM, BAD, BID, NOXA, PUMA, BMF, HRK and BIK only show homology to the BH3 domain of BCL-2. The effector proteins BAX, BAK and BOK contain multiple BH domains and structural studies of BAX revealed that effector protein three-dimensional conformation is similar to that of pro-survival BCL-2 proteins [39]. Like the pro-survival BCL-2 proteins, multiple pro-apoptotic proteins are found expressed in cells at the same time.

Upregulation of BH3-only proteins can occur at transcriptional/post-translational levels in response to stress to trigger cell death. For example, *Puma* and *Noxa* are transcriptional targets of the p53 tumour suppressor and their expression is increased in response to cytotoxic stimuli that activate p53, although PUMA is also important in response to p53-independent apoptotic stimuli [40]. BH3-only proteins can also be regulated by post-translational modification. Phosphorylation of BAD leads to sequestration by 14-3-3 proteins in the cytosol where it cannot exert pro-apoptotic functions [41]. Other mechanisms of activation exist such as altered cellular localization. Full-length BID is located in the cytosol but upon cleavage by caspase 8 (downstream of death-receptor signalling in the extrinsic apoptotic pathway) a truncated product is formed (tBID) which is capable of locating to the mitochondria and activating apoptosis [42].

BAX and BAK contain membrane anchoring C-terminal tails and while BAK is constitutively bound to the outer mitochondrial membrane, in healthy cells BAX appears cytosolic [43]. However, BAX and (to a lesser degree) BAK are actually in a dynamic equilibrium between cytosol and membranes, and are constitutively retrotranslocated to the cytosol by pro-survival BCL-2 proteins [44–46]. In the absence of any other BCL-2 proteins BAX becomes membrane localized, like BAK [45].

The mechanism of activation of BAX/BAK by BH3-only proteins has been the subject of intense debate. Evidence exists to suggest that some BH3-only proteins are ‘activators’: in this model BIM, tBID and PUMA, can directly interact with BAX/BAK to trigger their conformational change [47,48]. The function of the other BH3-only proteins in this model is as ‘sensitizers’, whose binding to BCL2-like pro-survival proteins frees up ‘activator’ BH3-only proteins [47,48]. An alternative, ‘indirect’ model has also been proposed whereby pro-survival BCL-2 proteins exert their function through direct interaction with BAX/BAK and BH3-only proteins act to sequester pro-survival proteins away from BAX/BAK. Such interactions do not always depend on BH3 domains as transmembrane domain (TMD) dimerization also occurs in the outer mitochondrial membrane. This has been observed in non-apoptotic cells and could indicate competition between the TMDs of pro-survival proteins such as BCL-2 and BCL-XL to prevent BAX/BAK homo-oligomerization [49].

Recently, genome editing was used to disrupt all known BH3-only proteins (eight in total) in HCT116 cells rendering these cells resistant to stress-induced apoptosis [50]. Interestingly, treatments that downregulate/target MCL-1- and BCL-XL-induced apoptosis with equivalent kinetics to those seen in cells proficient for BH3-only proteins revealing that known BH3-only proteins are not required for BAX/BAK activation [50]. Instead, association with the mitochondrial outer membrane is sufficient to drive homo-oligomerization of BAX/BAK in the absence of pro-survival BCL-2 proteins [50]. Regardless of which model is active in a particular cell type/situation, the outcome of a relative increase in the levels of pro- versus anti-apoptotic BCL-2 proteins is the same and the C-termini of BAX/BAK undergo conformational change that allows dimerization [51,52]. Reciprocal interaction between the BH3 domain of one molecule and the hydrophobic groove on another results in symmetrical homodimers (although heterodimers of BAX/BAK can also form in this manner) and linkage of these leads to higher-order oligomerization [53,54]. These oligomers delineate arcs, lines or ring-like structures in the outer mitochondrial membrane [55,56]. This mitochondrial outer membrane permeabilization (MOMP) allows release of soluble proteins from the mitochondrial inner membrane space such as cytochrome *c* which binds to APAF-1 (apoptotic protease activating factor 1), promoting its oligomerization and binding to pro-caspase 9—forming a complex termed the apoptosome. At the apoptosome, pro-caspase-9 is activated via dimerization, which in turn cleaves and activates the executioner caspases-3 and -7 to drive mass proteolysis that leads to DNA fragmentation, chromatin condensation and the dismantling of the cell. It is important to note that the mitochondrial pathway also contributes to death-receptor-mediated apoptosis through caspase 8 cleavage of BID, indeed BAX/BAK are required in many circumstances for receptor-mediated apoptosis [57,58].

*In vivo* analysis suggests the function of BAX and BAK is largely redundant in physiological settings; only a minority of double knockout mice survive to adulthood [59], but one copy of either is enough to allow normal development. In the absence of BAX/BAK apoptosis is impaired in response to almost all stimuli showing their requirement for the initiation of MOMP [60]. Less well studied, BOK is an additional multi-domain pro-apoptotic BCL-2 protein that was identified through its interaction with MCL-1 [61], although subsequent studies have suggested that BOK does not interact with pro-survival BCL-2 proteins [62,63]. BOK transcripts are present in many tissues in mice [64] but the protein is short-lived due to turnover by ubiquitylation and endoplasmic reticulum-associated degradation mediated by the proteasome [63].

BOK shows strong homology to BAX and BAK, and its role in apoptotic regulation has recently been extensively investigated. BOK deficient mice appear normal [64], but enforced BOK expression can drive apoptosis in a range of cell types and there is debate over whether this requires BAX/BAK [62–67]. Endogenous BOK is found predominantly in the membranes of the Golgi apparatus and endoplasmic reticulum (ER) and a function in ER stress response has been suggested [62,65]. More recently, the ability of proteasome inhibitors to induce apoptosis in BAX/BAK-deficient MEF or HCT116 cells was shown to require BOK expression but this was independent of pro-survival BCL-2 family proteins [63]. For now, the role of BOK certainly seems unique and requires further investigation.

## 5. Favoured interactions

**Figure 3.**
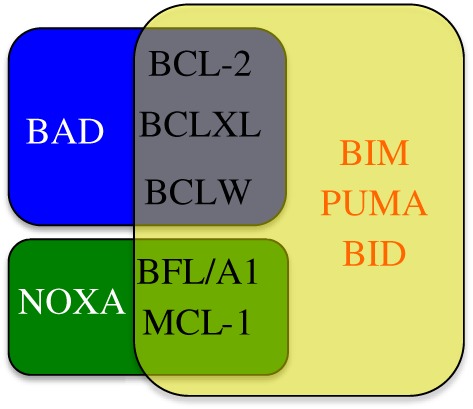
Specific interactions of BH3-only with pro-survival proteins. Some BH3-only proteins (BIM, PUMA and BID) are promiscuous and can bind all pro-survival BCL-2 proteins, whereas others (BAD and NOXA) show a more restricted binding pattern.

As discussed above, a level of specificity is granted by differential expression pattern, cellular localization, post-translational activation and turnover of BCL-2 family proteins. A further level of complexity was revealed by biochemical and cell biology studies that showed differential binding affinities of particular BH3-only proteins for pro-survivals [68,69]. While BH3-only proteins such as BIM, PUMA and tBID bind with high affinity to all pro-survival proteins, others are more selective in their interaction. For example, BAD preferentially interacts with BCL2, BCL-XL and BCL-W while NOXA showing a reciprocal interaction preference for only MCL-1 and A1 (figure 3). Further complexity is added by preference of BH3-only proteins for activation of BAX or BAK. While BIM and BID bind the same repertoire of pro-survival proteins (figure 3), preference of BID to mediate apoptosis through BAK has been shown [70,71], whereas BIM preference for BAX [70], or no preference [71], has been observed. Domain swap experiments have revealed that these effects are determined by the BH3 sequence of the BH3-only proteins and are dependent on cell type [71].

An additional layer of complexity is added with specificity in pro-survival proteins for BAK versus BAX activation. MCL-1 and BCL-XL constrain BAK, but BCL-2 does not [72], and it is the BH3 domain of BAK that determines these associations [71].

### 5.1. Increased pro-survival BCL-2 proteins in cancer

Evasion of apoptosis can aid oncogenic transformation at multiple stages through facilitating sustained tumour growth, survival during metastatic process and resistance to therapy. Therefore, it is not surprising that increased expression of pro-survival BCL-2 proteins is found in many cancer types. This upregulation can occur through a variety of mechanisms including chromosomal translocation, gene amplification, increased gene expression/translation or protein stability with various mechanisms and alternative pro-survival BCL-2 protein increases seeming more prominent in particular cancers.

Besides the *Bcl2* t(12;18) translocation, first found in follicular lymphoma (and subsequently in diffuse large-cell lymphomas [73]), translocation of BCL-2 family genes is not common across different cancer types nor does it seem to occur to other pro-survival members. Interestingly, t(14;18) translocation of *Bcl2* has also been found in peripheral blood lymphocytes from healthy individuals [74] and modelling of this translocation in B cells of mice is only weakly tumorigenic [75]. Together, these data suggest that this translocation is not overtly oncogenic.

**Figure 4.**
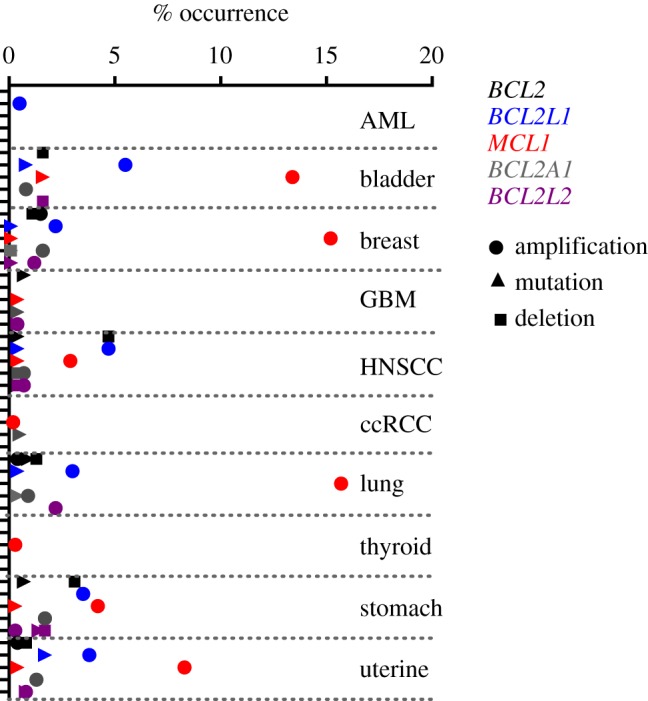
Frequency of genomic alteration of pro-survival BCL-2 proteins in cancer. Frequency of amplification (circle), mutation (triangle) or deletion (square) of pro-survival BCL-2 members in a range of cancers. Data mined from TCGA studies through cBioportal [77]. BCL-2 (black), BCL-XL (BCL2L1 blue), MCL-1 (red), BFL (BCL2A1 grey), BCL-W (BCL2L2 purple). AML, acute myeloid leukaemia [78], 173 cases. Bladder, urothelial carcinoma nature (TCGA provisional), 408 cases. Breast, invasive carcinoma (TCGA provisional), 1100 cases. GBM, glioblastoma [79], 166 cases. HNSCC, head and neck squamous cell carcinoma (TCGA provisional), 522 cases. ccRCC, kidney renal clear cell carcinoma [80], 534 cases. Lung adenocarcinoma (TCGA provisional), 517 cases. Thyroid, papillary thyroid carcinoma [81], 509 cases. Stomach adenocarcinoma (TCGA provisional), 415 cases. Uterine, corpus endometrial carcinoma [82], 177 cases.

Gene copy number increases in pro-survival BCL-2 members in cancer are more widespread than translocation. Transgenic modelling of increases in pro-survival proteins has mostly been limited to the haematopoietic systems where elevation of *Bcl2*, *Mcl1* or *Bclx* predisposes to lymphoma development, albeit with long latency and incomplete penetrance. Amplification of *MCL1* and *BCL2L1* (encodes BCL-XL) were found to be among the most frequent chromosomal gains in a study of over 3000 samples representing 26 tumour types [76]. It is interesting that these amplifications were prominent outside of haematopoietic cancers, which have been the traditional niche for studies on tumorigenic roles of the BCL-2 members. Indeed, analysis of The Cancer Genome Atlas (TCGA) data through cBioportal [77] confirms the prevalence of *MCL1*, and to a lesser extent *BCL2L1*, amplification in many solid cancers (figure 4). Again, there seems specificity between family members as *BCL2, BCL2A1* (BFL) and *BCL2L2* (BCL-W) amplification are much rarer events. Consistent with a pro-tumour role, mutation or deletion of pro-survival BCL2 proteins was also infrequent (figure 4). While pro-survival BCL-2 expression on its own is mildly oncogenic, acquisition of additional genetic hits is clearly required for tumour formation. Co-amplification of *MYC* with *MCL-1* or *BCLX* is common in cancer [76] and in cell culture and mouse models increased BCL-2-like proteins, and MYC is a potent oncogenic combination [83–87].

Increased transcription of pro-survival proteins can also elevate their expression in cancer and increased transcription/translation seems important for regulation of MCL-1 levels. For example, in chronic lymphocytic leukaemia (CLL) c-ABL has been shown to drive high MCL-1 mRNA and protein expression through STAT3/NF-κB [88]. Similarly, in a mouse model of B-cell acute lymphoblastic leukaemia (B-ALL) the BCR-ABL oncoprotein was shown to drive high levels of MCL-1 expression that was essential for leukemogenesis [89].

Increased protein translation impacts on MCL-1 and BCL-XL levels and MCL-1 has been identified as a downstream mediator of the oncogenic effect of the translation initiation factor eIF4e [90]. Disruption of pathways regulating MCL-1 protein stability also occurs in cancer (e.g. loss or mutation of FBW7 inhibits MCL-1 degradation and is associated with tumorigenesis and resistance to chemotherapy [91,92]). Therefore, in addition to genetic alterations in BCL-2 family members, activation of oncogenic signalling pathways can also increase pro-survival protein levels.

It is important to consider that the observation of high levels of pro-survival BCL-2 proteins in cancer need not necessarily indicate strong apoptotic resistance. Elevation of BCL-2 actually sensitizes to apoptosis induced by the BCL-2/BCL-XL/BCL-W targeting drug ABT-737 through release of high levels of BIM that have been harboured in BIM/BCL-2 complexes [93]. In this way, a cell can be thought of as primed for death, close to the threshold required for apoptosis induction and measurement of the level of mitochondrial priming in cells can be used to predict response to chemotherapy [94,95].

Alterations in pro-survival BCL2 proteins might have importance beyond cancer genesis as high levels of MCL-1 expression at diagnosis correlate with poor prognosis in breast cancer [96], and MCL-1 amplification is prominent in treatment-resistant breast cancers [97], suggesting that this may be a source of innate and acquired resistance to cancer therapy. Such associations do not hold true for pro-survival BCL-2-like proteins in general and high levels of BCL-2 are actually associated with good prognosis in breast cancer [98–101].

### 5.2. Decreased pro-apoptotic BCL-2 proteins in cancer

Decreased expression of pro-apoptotic BCL-2 proteins has the same functional outcome as increased pro-survival expression in cancer. Gene deletion studies in mice do not reveal a strong oncogenic impact of decreased BH3-only protein expression. With the exception of BAD knockout mice, which succumb to late onset lymphoma [102], deficiency in individual BH3-only proteins does not predispose mice to tumour development [103]. Functional redundancy between BH3-only proteins could account for this, however, compound deletion of multiple BH3-only proteins is still only weakly tumour promoting with autoimmunity contributing to morbidity [104].

Similar to over-expression of pro-survival BCL2-like proteins, loss of BH3-only proteins is not overtly oncogenic but can dramatically accelerate lymphoma development in the context of elevated MYC. BIM seems most potent in this context, with deletion of even a single allele of BIM having dramatic effects [105].

Cancer therapeutics engaging the p53 response would be predicted to upregulate PUMA and NOXA and resistance to apoptosis could be mediated by their downregulation. Indeed, deletion of the gene encoding PUMA (*Bbc3*) occurs in a range of cancer types [76] and other mechanisms can decrease PUMA expression such as promoter methylation [106].

### 5.3. Altered expression of effector proteins

Elimination or downregulation of apoptotic effector proteins such as BAX/BAK is a potent way to disable mitochondrial-mediated apoptosis in cell culture systems. As the function of BAX and BAK are largely redundant, with one copy of either allowing normal development, abrogation of their activity would require loss of all four alleles. There is limited evidence that this occurs in cancer and BAX/BAK levels naturally decline with age in mice and humans [107]. It is important to note that localization and conformation may be even more important than absolute levels of BAX/BAK. For example, in acute myeloid leukaemia (AML) mitochondrial localized BAX is associated with both increased apoptotic sensitivity and improved patient prognosis [108]. Gene deletion studies in mice do not suggest a tumour suppressive role for BAX or BAK when knocked out individually (presumably due to their redundant roles) and double knockout results in perinatal lethality [59,109]. Using chimeric mice *Bax/Bak* deletion in the haematopoietic compartment drives fatal autoimmune disease [110]. The prominence of autoimmune disease in chimeric mice with *Bax/Bak* [111] deficient haematopoietic systems may mask tumour development. Targeted deletion mouse studies can avoid these autoimmune complications and in this setting loss of BAX/BAK in the brain and testicles results in tumours [112].

It seems pertinent loss of *Bok* is a relatively frequent event in a range of cancers [76] and gene silencing may result in loss of BOK protein in additional cases [111]. BOK is downregulated in non-small cell lung cancer (NSCLC) and high BOK is associated with good prognosis in lymph-node positive patients [111]. Interestingly, the tumour suppressive effect of BOK in NSCLC does not seem to be through apoptotic regulation and is instead through antagonism of TGF-β2 mediated epithelial to mesenchymal transition (EMT) and cell migration [111]. Unlike so many other BCL-2 proteins, *Bok* loss failed to reveal a tumour suppressive role the Eu-Myc mouse model [64], a system that is a sensitive read-out for altered apoptosis, although factors such as cooperating second hits, timing of *Bok* loss and cell type specificity could all be involved in a BOK effect. Compound knockout of *Bax/Bak/Bok* in the haematopoietic system also results in autoimmune disease rather than tumourigenesis [113]. More recently, in a chemical (DEN)-induced model of liver carcinogenesis, loss of BOK has been shown to protect against cancer. In this model, cancer can be promoted by death of hepatocytes, supporting compensatory proliferation with associated mutagenesis, in surrounding tissue that ultimately leads to live cancer [114]. Interestingly, deletion of BOK inhibited the ER-stress response and induction of pro-apoptotic BH3-only proteins BIM and PUMA, placing BOK's tumour promoting role upstream of MOMP [114]. Secondly, BOK was also shown to have an additional growth-promoting effect, though the mechanisms underlying this remain unclear it would also be expected to be pro-tumourigenic [114].

### 5.4. Pharmaceutical intervention to reset the balance

As the balance of BCL-2 proteins can act as a trigger for apoptosis, mechanisms to alter their expression or interaction would be expected to have clinical use. Indeed conventional anti-cancer therapies can act through disruption of the BCL-2 family. For example, DNA damaging agents such as etoposide or daunorubicin activate the p53 tumour suppressor transcription factor whose targets include PUMA and NOXA. However, the presence of elevated pro-survival BCL-2 proteins can act as a barrier to apoptosis even upon upregulation of BH3-only proteins. Neutralization of pro-survival proteins would re-sensitize to BH3-only upregulation and could even have the potential to trigger apoptosis alone.

**Figure 5.**
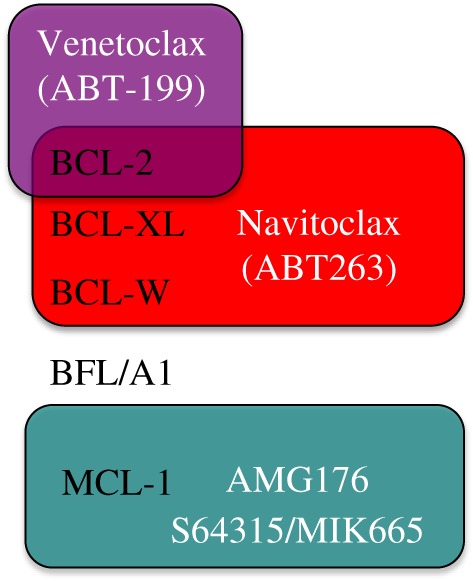
Selectivity of BH3-mimetic drugs under clinical investigation. Drugs specifically targeting BCL-2 (Venetoclax/ABT-199), BCL-2, BCL-XL and BCL-W (Navitoclax/ABT263) or MCL-1 (AMG176, S64315/MIK665) are now in clinical trial/use.

Interest in the development of inhibitors of pro-survival BCL-2 proteins has come to fruition over the past decade following the identification of small molecule inhibitors capable of occupying the hydrophobic pocket on pro-survival proteins. The first of these BH3 mimetic drugs, ABT-737, was developed through NMR-based fragment screening. ABT-737 mimics the BH3-domain of pro-apoptotic BAD and interacts with BCL-2, BCL-XL and BCL-W (figure 5), displacing BH3-only proteins to trigger apoptosis in cell lines and restrict tumour growth in xenograft models [2]. Derivation of an orally available analogue (ABT 263/Navitoclax) allowed clinical testing [115]. On-target toxicity of Navitoclax was observed with dose-limiting thrombocytopenia occurring due to platelet dependence on BCL-XL [116,117]. This can be managed in the clinic and preclinical models have supported the use of Navitoclax in clinical trials as a combination therapy in a range of (predominantly solid) tumour types [118,119] (clinicaltrials.gov).

Further development has led to additional BH3 mimetics with increased specificity for individual BCL-2 proteins. The success of this approach has been shown with the BCL-2 specific BH3 mimetic ABT-199 (Venetoclax), which obtained breakthrough FDA status for use in relapsed/refractory CLL. In this disease single-agent efficacy was seen with partial response in 79% and complete response in 20% of patients [120]. Even more impressive results were seen when Venetoclax was used in combination with rituximab-complete response occurred in 51% of patients with disease-free status occurring for up to 2 years after completion of therapy [121]. Encouraging (but more modest) effects are seen in non-Hodgkin lymphomas, acute myeloid leukaemia and multiple myeloma as a single agent [122–124] or combination therapy [125]. While much is understood of the role of the BCL-2 family in cancers of the blood, the case for targeting these proteins in solid tumours, most probably in conjunction with conventional therapies, is compelling. Translocation or amplification of *BCL2* itself is rarely seen in solid tumours but dependence on BCL2 has been shown in small cell lung cancer (SCLC) [126]. Efficacy of Venetoclax when used as a combination therapy has been shown in preclinical models of breast cancer [127] and Venetoclax is now in clinical trials in combination with tamoxifen in breast cancer (ISRCTN98335443).

Resistance to BCL-2 and BCL-2/BCL-XL targeting BH3 mimetics has been observed, and *in vitro* studies indicate that ABT-737 treatment can increase MCL-1 levels and MCL-1 expression promotes resistance to ABT737 *in vitro* and *in vivo* [128–130]. MCL-1 has been associated with resistance to cancer therapy for some time [131,132], and interest has intensified in the development of drugs that specifically target MCL-1. A number of MCL-1 inhibitors have been mooted but until very recently compounds with clear specificity and on-target effect were lacking [133]. The tide has turned and a number of robust BH3 mimetic drugs targeting MCL-1 are now available; Abbvie's A1210477 potently inhibits MCL-1 *in vitro* to restrict growth of diverse cancer cell lines [134,135], UMI-77 inhibits pancreatic and breast cancer cell line growth *in vitro* and *in vivo* [96,136], and the Servier compound S63845 seems particularly potent, with on-target single agent killing of leukaemia and lymphoma models *in vitro* and *in vivo* [4] and in combination with conventional cancer therapy in xenograft models of breast cancer [137]. The potential for MCL-1 specific BH3-mimetics in the clinic is now being tested with the Novartis/Servier drug S64315/MIK665 and Amgen AMG176 in phase I clinical trials for haematopoietic cancers/myelodysplastic syndrome (NCT02992483, NCT02979366, NCT02675452). Taking advantage of the short half-life of MCL-1 protein, a number of other approaches can be taken to decrease MCL-1 levels. This includes inhibition of transcription through CDK inhibition [138] or targeting translation through mTOR inhibition [139]. Beyond targeting anti-apoptotic BCL-2 proteins, increasing interest has centred on developing drugs that directly activate BAX and BAK in order to kill tumour cells [140,141]. Along these lines, a recent study has shown that a BAX-activating molecule, BTSA1, shows potent anti-tumour effects on human acute myeloid leukaemia (AML) xenografts in the absence of toxicity [142].

## 6. Inhibiting BCL-2 proteins in cancer prophylaxis

It is conceivable that targeting the pro-survival BCL-2 proteins could help eliminate pre-cancerous lesions or early-stage tumours. Indeed, ABT737 can act as an anti-cancer prophylactic in the Eμ-MYC mouse model of B-cell lymphoma, which has been shown to be dependent on BCL-XL [143]. Evidence for applicability beyond MYC-driven lymphoma is limited and in p53 null mice (which predominantly succumb to thymic lymphoma) prophylactic treatment with ABT737 had no impact on tumorigenesis [144]. When low-dose γ-irradiation was added to this experimental protocol to mimic environmental factors that could induce additional mutations prophylactic ABT737 treatment was shown to delay lymphoma onset in p53 null mice, but this reduction in thymic lymphoma was accompanied by increased incidence of sarcoma, and while significant, the difference in survival outcome afforded by prophylactic ABT 737 treatment was minimal [144].

Confounding factors include the altered activity of cells that have failed to be eliminated by targeting pro-survival BCL2 proteins. For example, the spontaneous apoptosis induced by targeted deletion of *mcl1* in hepatocytes results in severe liver damage and increased proliferation that actually results in hepatocellular carcinoma (HCC) [145]. Such effects have also been shown in models of γ-irradiation induced thymic lymphoma where deletion of *puma* or over-expression of *Bcl2* (normally considered as pro-cancer events) actually protects against tumour development [146]. In this scenario, γ-irradiation no longer causes depletion of bone marrow leucocytes meaning that there is no niche for the proliferation of stem/progenitor cells carrying damaged DNA that normally give rise to the thymic lymphomas in this model [146,147]. There are further indications that inhibiting the pro-survivals may not always have anti-cancer impact, in settings where ABT737 fails to induce apoptosis detrimental side effects can occur through activation of CAD and genome instability [148]. It remains to be seen whether these concerns hold true in the clinic.

In the decades following the discovery of BCL-2 (and related family members) a vast quantity of research has unravelled their role in regulating apoptosis. The functional division of this family into pro- and anti-apoptotic members and the elucidation of their structures and mechanism of interaction has allowed the pharmaceutical development of molecules to specifically inhibit these protein–protein interactions and reinstate apoptosis. Available data from clinical trials suggests good efficacy of BH3-mimetics targeting BCL-2 in some types of blood cancer. Deregulation of BCL-2 proteins is now recognized as a frequent event in many types of cancer and it seems likely that targeting pro-survival BCL-2 proteins will form a valuable adjunct to current cancer therapies. Restoration of apoptosis offers the potential to eliminate cancer cells at all stages of pathology and as BH3-mimetic drugs make their way into the clinic they could make dramatic improvements in survival outcome in cancer.

## Data accessibility

This article has no additional data.
